# Crystal Structure of Human Protein N-Terminal Glutamine Amidohydrolase, an Initial Component of the N-End Rule Pathway

**DOI:** 10.1371/journal.pone.0111142

**Published:** 2014-10-30

**Authors:** Mi Seul Park, Eduard Bitto, Kyung Rok Kim, Craig A. Bingman, Mitchell D. Miller, Hyun-Jung Kim, Byung Woo Han, George N. Phillips

**Affiliations:** 1 Research Institute of Pharmaceutical Sciences, College of Pharmacy, Seoul National University, Seoul, Korea; 2 Department of Chemistry and Biochemistry, Georgian Court University, Lakewood, New Jersey, United States of America; 3 Department of Biochemistry, Center for Eukaryotic Structural Genomics, University of Wisconsin Madison, Madison, Wisconsin, United States of America; 4 Department of Biochemistry and Cell Biology, Rice University, Houston, Texas, United States of America; 5 College of Pharmacy, Chung-Ang University, Seoul, Korea; Yale University School of Medicine, United States of America

## Abstract

The N-end rule states that half-life of protein is determined by their N-terminal amino acid residue. N-terminal glutamine amidohydrolase (Ntaq) converts N-terminal glutamine to glutamate by eliminating the amine group and plays an essential role in the N-end rule pathway for protein degradation. Here, we report the crystal structure of human Ntaq1 bound with the N-terminus of a symmetry-related Ntaq1 molecule at 1.5 Å resolution. The structure reveals a monomeric globular protein with alpha-beta-alpha three-layer sandwich architecture. The catalytic triad located in the active site, Cys-His-Asp, is highly conserved among Ntaq family and transglutaminases from diverse organisms. The N-terminus of a symmetry-related Ntaq1 molecule bound in the substrate binding cleft and the active site suggest possible substrate binding mode of hNtaq1. Based on our crystal structure of hNtaq1 and docking study with all the tripeptides with N-terminal glutamine, we propose how the peptide backbone recognition patch of hNtaq1 forms nonspecific interactions with N-terminal peptides of substrate proteins. Upon binding of a substrate with N-terminal glutamine, active site catalytic triad mediates the deamination of the N-terminal residue to glutamate by a mechanism analogous to that of cysteine proteases.

## Introduction

Aberrant polypeptides or proteins should be accurately removed in many physiological processes. Intracellular protein degradation is mainly conducted through the ubiquitin-proteasome pathway (UPP) or the lysosomal proteolysis [1]. The UPP is required for degradation of short-lived proteins in eukaryotic cells. In the UPP, ubiquitin first attaches to target proteins or polypeptides, which leads to their recognition by the 26S proteasome [2]. On the other hand, lysosomal proteolysis leads to breakdown of unnecessary proteins or polypeptides by lysosomes [3].

The N-end rule is related to the ubiquitin-dependent proteolytic system [4]. The N-end rule is one of common pathways for the degradation of polypeptides and proteins in prokaryotes and eukaryotes, which determines the stability of a protein by its N-terminal residue [1]. Val, Gly, and Pro are classified as stabilizing residues in mammals whereas Asp, Gln, Cys, and Arg are known as destabilizing residues [5]. In the N-end rule pathway, N-terminal glutamine and asparagine are tertiary destabilizing residues and these residues are converted into secondary destabilizing N-terminal glutamate and aspartate by deamidation [6], [7]. Arginine is then conjugated to glutamate and aspartate residues by Arg-tRNA-protein transferase, converting target proteins into ones possessing a primary destabilizing residues [6]–[8]. The N-end rule mechanism is involved in degradation of misfolded proteins, regulation of DNA repair, apoptosis and meiosis [9]–[12]. The N-terminal amidohydrolases are classified into the N-terminal glutamine amidohydrolase (Ntaq) and the N-terminal asparagine amidohydrolase (Ntan), which share low amino acid sequence identity and mediate the deamidation of N-terminal glutamine and asparagine, respectively [6].

In this work, we present the crystal structure of hNtaq1 bound with the N-terminus of a symmetry-related Ntaq1 molecule at 1.5 Å resolution. The structure contains the catalytic triad (Cys-His-Asp) in the active site, which is well conserved among Ntaq proteins and transglutaminases from diverse organisms. Additionally, we conducted docking study with all the tripeptides containing N-terminal glutamine to elucidate how N-termini of proteins with N-terminal glutamine are recognized and positioned by hNtaq1 into catalytically conducive conformations. We also propose a catalytic mechanism of hNtaq1 based on the crystal structure of hNtaq1 and docking study.

## Materials and Methods

### Cloning, protein expression, and purification

The standard Center for Eukaryotic Structural Genomics pipeline protocols were used for cloning [13], protein expression [14], protein purification [15]. In summary, using Gateway cloning (Life Technologies, USA), hNtaq1 gene was cloned into pVP16 plasmid (Clontech, USA) containing N-terminal fusion (His)_6_-Maltose Binding Protein (MBP) and a linker region with the TEV protease site for cleavage of target proteins. It results in the hNtaq1 construct mutated with Ser for the initial Met. The hNtaq1 construct was transformed into B834 *E. coli* cells (Novagen, USA) to express Se-Met labeled protein. Cells were cultured with auto-induction medium adapted from the work of Studier [16] and incubated in a shaker at 250 rpm, 25°C for 22∼24 hours. Cells were harvested by centrifugation at 5000×g for 20 min and suspended in cell lysis buffer (20 mM sodium phosphate, pH 7.5, 500 mM sodium chloride, 20% ethylene glycol, 35 mM imidazole, 0.3 mM tris(2-carboxyethyl) phosphate (TCEP), and E64 protease inhibitor cocktail (Sigma Aldrich, USA). Cells were disrupted by sonication on ice and the cell lysate was centrifuged at 75,600×g for 30 min twice. The supernatant was collected and filtered through a 0.8 µm pore size filter.

The sample was loaded on the Ni-charged HiTrap chelating 5 ml HP column (GE Healthcare, UK) and washed with IMAC-washing buffer (20 mM sodium phosphate, pH 7.5, 500 mM sodium chloride, and 0.3 mM TCEP). The protein was eluted by applying a 30 column volume linear gradient from 10% to 80% IMAC-elution buffer (20 mM sodium phosphate, pH 7.5, 350 mM imidazole, 500 mM sodium chloride, and 0.3 mM TCEP) and buffer was exchanged to TEV proteolysis buffer (20 mM sodium phosphate, pH 7.5, 100 mM sodium chloride, and 0.3 mM TCEP) using a HiPrep 26/10 desalting column (GE Healthcare, UK). The (His)_6_-MBP fusion hNtaq1 protein was treated with TEV protease (1∶100 w/w) at 25°C for overnight. After the TEV protease treatment, the protein was loaded to HiTrap chelating 5 ml HP column (GE Healthcare, UK) and eluted with IMAC-washing buffer. The eluted sample was desalted with crystallization buffer (50 mM sodium chloride, 3 mM sodium azide, 0.3 mM TCEP, and 100 mM Bis-Tris, pH 7.0) using a HiPrep 26/10 desalting column (GE Healthcare, UK). For crystallization, the purified Se-Met hNtaq1 protein was concentrated to 10 mg/ml.

### Crystallization, X-ray data collection, structure determination, and model evaluation

Crystals of the hNtaq1 were obtained by hanging-drop vapor diffusion method at 291 K by mixing the protein solution (10 mg/ml Se-Met protein, 50 mM sodium chloride, 3 mM sodium azide, 0.3 mM TCEP, and 100 mM Bis-Tris, pH 7.0) and the well solution (1% ethylene glycol, 1.8 M ammonium sulfate, 100 mM MES, pH 6.0) in 1∶1 ratio. Crystals were cryoprotected in four stages with well solution using containing 0 to 25% ethylene glycol and were flash-frozen in liquid nitrogen gas at 100 K. X-ray diffraction data were collected using synchrotron beam line 23-ID-D at the Advanced Photon Source of the Argonne National Laboratory. The crystal structure of the hNtaq1 was solved by SAD phasing at 1.5 Å resolution. SHARP [17] was used to solve experimental phase information, which was improved by density modification using DM [18]. Crystals of hNtaq1 belong to the space group *P*2_1_2_1_2_1_ with unit cell parameters a = 34.3 Å, b = 64.0 Å, and c = 113.6 Å. Subsequent manual model building and refinement were carried out using *Coot* [19] and *REFMAC* [20] from CCP4 program suite [20]. All refinement steps were monitored using an *R*_free_ value based on 5.0% of the independent reflections. The stereochemical quality of the final model was assessed using PROCHECK [21] and *MolProbity* [22]. The data collection, phasing, and refinement statistics are summarized in Table 1.

**Table 1:** Statistics for data collection, phasing, and model refinement.

Data collection and phasing^a^	
Space group	*P* 2_1_ 2_1_ 2_1_
Cell dimensions	
a, b, c (Å), α, β, γ (°)	34.32, 64.04, 113.66, 90, 90, 90
Data set	Se λ1 (peak)
X-ray wavelength (Å)	0.9794
Resolution range (Å)^b^	32.86–1.50 (1.53–1.50)
<*I*/σ(*I*)>	12.1 (2.5)
Multiplicity	12.2 (6.9)
Unique reflections	40,943 (2,566)
Completeness (%)	99.5 (95.9)
*R_merge_* (%)^c^	0.5 (54.3)
Figure of merit^d^ for SAD phasing: 0.44	
**Refinement**	
*R_work_* e/*R_free_* f	0.144/0.170
No. of protein atoms	1,666
No. of water atoms	332
No. of Non-water atoms	75
Mean B value (Å^2^)	18.9
Ramachandran plot analysis (for Chain A)	
Most favored regions	198 (96.6%)
Additional allowed regions	7 (3.4%)
Disallowed regions	0 (0%)
R.m.s. deviations from ideal geometry	
Bond lengths (Å)	0.017
Bond angles (°)	1.91

a Data collected at the Sector 23-ID-D of the Advanced Photon Source.

b Numbers in parentheses indicate the highest resolution shell of 20.

c *R_merge_* = Σ_h_ Σ_i_ |*I*(*h*)_i_–<*I*(*h*)>|/Σ_h_ Σ_i_ *I*(*h*)_i_, where *I*(*h*) is the observed intensity of reflection h, and <*I*(*h*)> is the average intensity obtained from multiple measurements.

d Figure of merit = <|Σ P(α)e^iα^/Σ P(α)|>, where α is the phase angle and P(α) is the phase probability distribution.

e *R_work_* = Σ | |*F_o_*|–|*F_c_*| |/Σ |*F_o_*|, where |*F_o_*| is the observed structure factor amplitude and |*F_c_*| is the calculated structure factor amplitude.

f *R_free_* = R-factor based on 5.0% of the data excluded from refinement.

### Docking studies of tripeptides with N-terminal glutamine

AutoDock Vina program [23] was used for the docking studies of hNtaq1 with all the possible 400 tripeptides containing N-terminal glutamine (Gln-X-X, X is any amino acid residue, thus 20×20 candidates). Coordinatees for the 400 tripeptides were generated using Coot [19] and converted to pdbqt files using AutoDockTools4 [24]. The grid maps for docking studies were centered on C_α_ of Ser1 of the bound N-terminus from the symmetry-related hNtaq1 molecule in the substrate binding cleft and the maps comprised 30×30×30 points. AutoDock Vina program was run with four-way multithreading and the default settings were used for others computational parameters. Figures are generated using PyMol [25].

### Data deposition

Atomic coordinates and structure factors have been deposited in the RCSB Protein Data Bank, accession code 4W79.

## Results and Discussion

### Overall structure of hNtaq1

**Figure 1:**
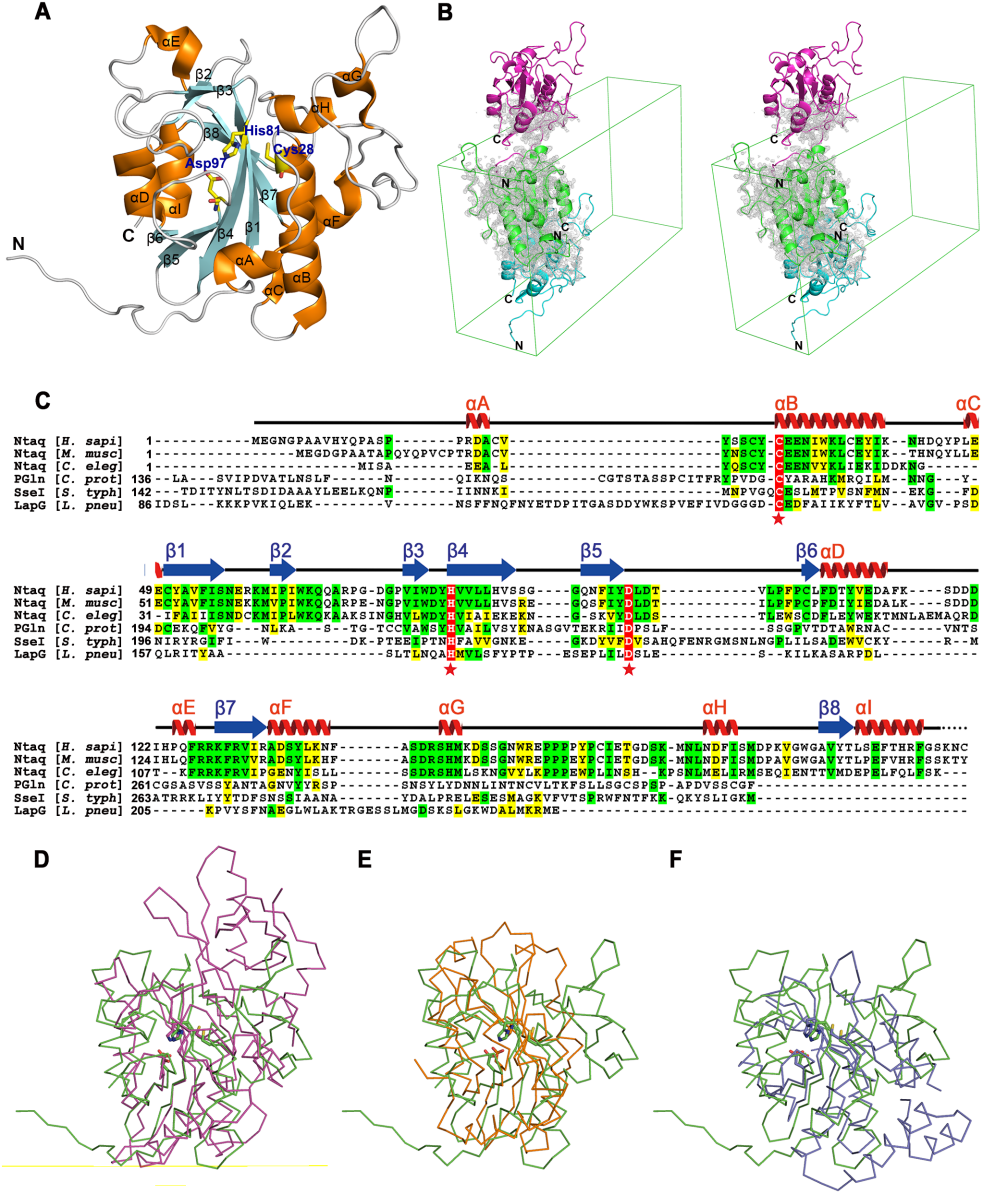
Overview of the crystal structure of hNtaq1. (A) Overall structure of hNtaq1. α-helices, β-strands, and loops are colored in orange, cyan, and white, respectively. The representative amino acid residues in the active site are shown as stick model (carbon, oxygen, nitrogen, and sulfur in yellow, red, blue, and gold color, respectively). (B) Stereo view of the crystallographic contact of hNtaq1 with a symmetry-related molecule. hNtaq1 and symmetry-related molecules are represented as green, cyan, and magenta cartoon, respectively. Unit cell is shown with green line and electron density map is shown as gray cloud. (C) Sequence alignment of hNtaq1 with Ntaq proteins from *Mus musculus*, *Caenorhabditis elegans*, protein-glutaminase from *Chryseobacterium proteolyticum*, secreted effector protein SseI from *Sallonella typhimurium*, and periplasmic protease LapG from *Legionella pneumophila*. Residues of the catalytic triad are represented by red asterisk below the amino acid sequences. Completely conserved, identical, moderately conserved residues are highlighted with red, green, and yellow shaded boxes, respectively. The secondary structure of hNtaq1 is shown on top of the sequence alignment. α-helix, β-sheet, and connecting region are represented by red spiral, blue arrow, and black line, respectively. Structural comparison of hNtaq1 with protein-glutaminase from *C. proteolyticum* (D), secreted effector protein SseI from *S. typhimurium* (E), and periplasmic protease LapG from *L. peumophila* (F). The catalytic triads are shown as stick models and main chains of hNtaq1, protein-glutaminase, SseI, and LapG are represented with ribbon diagram in green, magenta, orange, and blue, respectively.

The human C8orf32 gene encodes human N-terminal glutamine amidohydrolase isoform 1 (hNtaq1). Ntaq is an initial component of the N-end rule pathway and converts N-terminal glutamine to glutamate. In order to understand the relationship between the structure and function of hNtaq1, we determined the crystal structure of recombinant hNtaq1 bound with the adjacent N-terminus of a symmetry-related hNtaq1 molecule at 1.5 Å resolution. The hNtaq1 protein contains 205 amino acids, of which 202 have been successfully modeled in the presented hNtaq1 structure. Three C-terminal residues (Lys203, Asn204, and Cys205) were disordered in the crystal and could not be modeled. The *R* and *R*_free_ values of the final refined model were 14.4% and 17.0%, respectively. hNtaq1 is a monomeric globular protein with a novel structural fold of alpha-beta-alpha three-layer sandwich architecture (Figure 1A). To our surprise, the N-terminus of a symmetry-related hNtaq1 molecule was captured in the substrate binding cleft, even though Ser1 is the N-terminal residue, not an anticipated glutamine residue (Figure 1B). The core region of the protein shows antiparallel beta-sheets surrounded by helices. The catalytic triad (Cys28, His81, and Asp97) is highly conserved among Ntaq proteins, transglutaminases, and cysteine proteases of diverse organisms (Figure 1C). Ntaq and Ntan in human share only 13.1% sequence identity and the structure of Ntan has yet not been determined. Elucidation of subtle differences in the catalytic mechanism between Ntaq and Ntan in the N-end rule pathway awaits the structural information of Ntan.

To find out structural features of hNtaq1, we analyzed of structure similarity using the DALI server [26]. The result reveals that hNtaq1 shows low level of sequence conservation with other structurally similar proteins and the amino acid sequence identities are in the range of 9 to 15%. The result shows that protein-glutaminase from *Chryseobacterium proteolyticum* (PDB ID: 3A54) is the closest structural homolog of hNtaq1 (Z score 7.0 and RMSD distance 3.6 Å for 123 equivalent C_α_ positions out of 280 residues) and shares only 15% sequence identity. The protein-glutaminase from *Chryseobacterium proteolyticum* converts a glutamine residue to a glutamate and has catalytic triad comprising Cys156, His197, and Asp217 [27] (Figure 1D). The secreted effector protein SseI from *Sallonella typhimurium* (PDB ID: 4G2B) is the second best structural homolog of hNtaq1 (Z score 6.3 and RMSD distance 3.7 Å for 116 equivalent C_α_ positions out of 169 residues) and shares only 9% sequence identity. The secreted effector protein SseI from *Sallonella typhimurium* belongs to cysteine protease superfamily and also contains catalytic triad with Cys178, His216, and Asp 231 [28] (Figure 1E). LapG from *Legionella pneumophila* (PDB ID: 4FGP), a bacterial transglutaminase-like cysteine protease (BTLCP) containing catalytic triad, also shares structural similarity to hNtaq1 (Z score 6.1 and RMSD distance 2.7 for 96 equivalent C_α_ positions out of 186 residues) [29] (Figure 1F). Even though structural homologs of hNtaq1 do not share high sequence identities, the catalytic triad is structurally well conserved, mediating similar type of reactions with different substrates. Uniqueness of substrate binding region except the catalytic triad could contribute to the specificity with respect to each substrates.

### Active site with catalytic triad of hNtaq1

**Figure 2:**
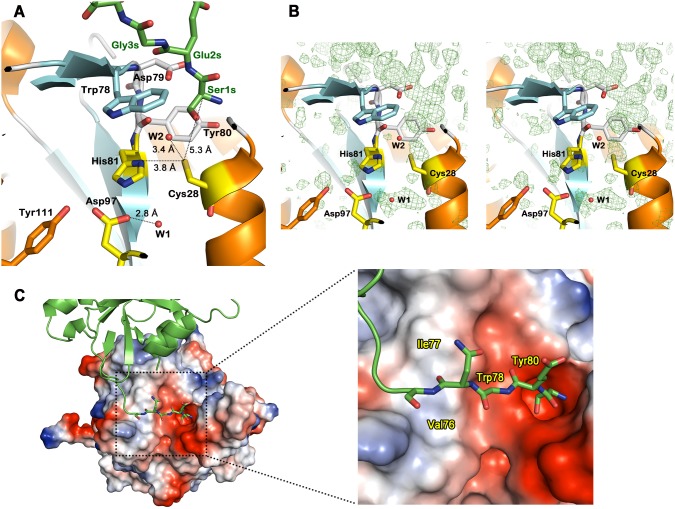
Active site and electrostatic potential surface charge of hNtaq1. (A) Substrate binding cleft of hNtaq1. Carbon in the substrate-mimicking peptide, catalytic triad, α-helices, β-strands, and loops are colored in green, yellow, orange, cyan, and white, respectively. Oxygen, nitrogen, and sulfur atoms are represented as red, blue, and gold, respectively. Two water molecules are shown as red sphere and labeled as W1 and W2. (B) Electron density map from an *Fo*–*Fc* omit map calculated without the bound substrate-mimicking peptide. Positive electron density are shown as a green mesh contoured at 2.0 σ, in a stereo view. (C) Electrostatic potential surface and substrate binding cleft region of hNtaq1. Negatively and positively charged surfaces are represented as red and blue shade, respectively. Residues interacting with the substrate-mimicking peptide molecule are labeled.

The structure of hNtaq1 shows the precise conformation of the active site and the catalytic triad, Cys28, His81, and Asp97. Previous researches showed that Cys and His residues play a vital role in the activity of Ntaq. In the case of mouse Ntaq Cys30Ala and His83Ala mutants, the mutations almost abolish activities as an N-terminal glutamine amidohydrolase [8]. In the structure of hNtaq1, Asp97 forms hydrogen bonds with His81, Tyr111 and a water molecule 1 (water403 in PDB) and His81 interacts with another water molecule 2 (water444 in PDB) via hydrogen bond. The distances from the sulfhydryl group of Cys28 to water2 and His81 are 3.4 Å and 3.8 Å, respectively. After proper conformational changes that shorten the distance between His81 and Cys28 during a catalytic conversion, the sulfhydryl group of Cys28 is deprived of proton and increases its nucleophilicity for a successive attack on substrates (Figure 2A).

Interestingly, the crystal structure of hNtaq1 revealed an unusual crystallographic contact between symmetry-related molecules. The N-terminus of a symmetry-related monomer is anchored into the active site of hNtaq1 with the side chain of the N-terminal residue (Ser1) in close proximity of the catalytic Cys28. The electron density of the bound N-terminus of a symmetry-related monomer could be clearly observed in an *F_o_*–*F_c_* omit map when calculated without the N-terminus residues as shown in Figure 2B. This phenomenon was similarly observed in the case of crystal structure of another N-end rule pathway protein, UBR box of ubiquitine ligases (PDB ID: 3NIS) [30]. The N-terminus of the symmetry-related monomer (from now on, the “substrate-mimicking” peptide) forms an antiparallel beta-strand segment that adheres to the surface exposed beta-strand of hNtaq1 (residues 76–79, β3 in Figure 1A). The strands are stabilized by several interactions: 1) three peptide backbone hydrogen bonds typical for beta-sheet structures (two between Ile77 and Asn4s, and one between Asp79 and Glu2s); 2) a hydrogen bond between side chain of Asp79 and peptide backbone of the N-terminal residue Ser1s; 3) a hydrogen bond between Tyr80 and the amino group of the substrate-mimicking peptide; 4) a direct van der Waals contact of Trp78 with the substrate-mimicking peptide. The surface charge of the binding cleft is predominantly negative, and several hydrophobic and aromatic residues (Val76, Ile77, Tryp78, and Tyr80) are well oriented to recognize main chain backbone of the substrate-mimicking peptide and to stabilize aliphatic part of the bound substrate-mimicking peptide (Figure 2C). Overall, the active site bound with the substrate-mimicking peptide directly suggests possible interaction mode of hNtaq1 with substrates.

### Docking study with anticipated substrate peptides

**Figure 3:**
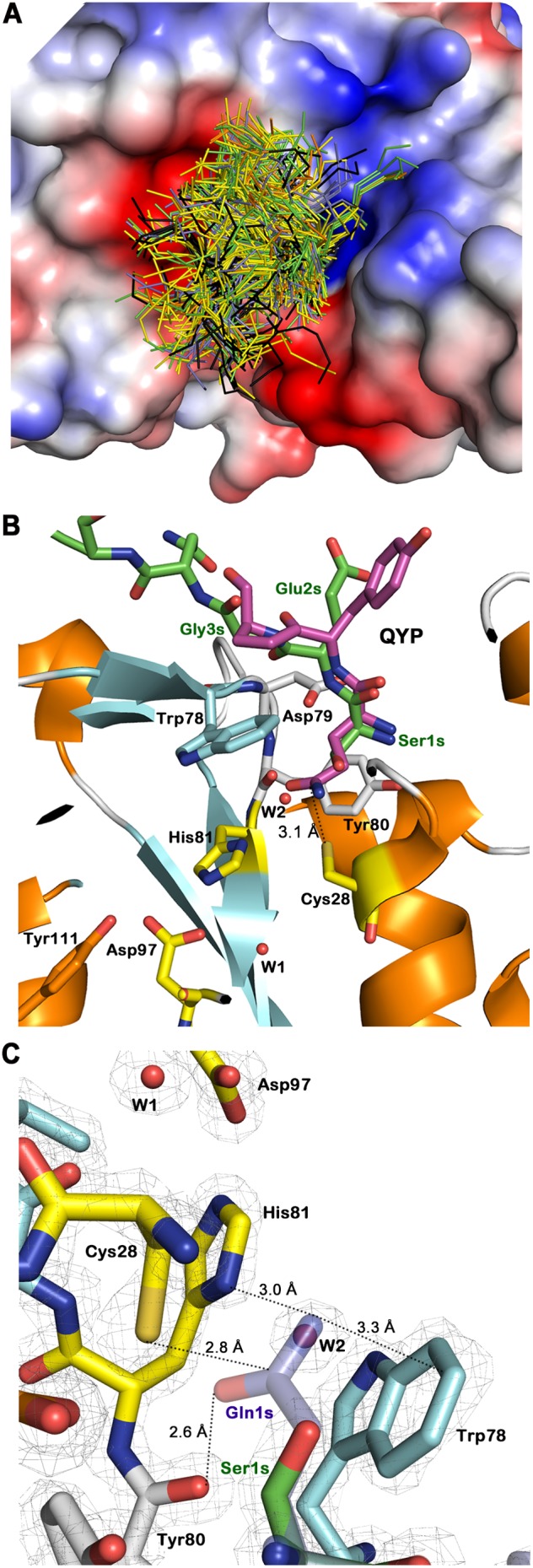
Docking study and suggested binding pose of N-terminal glutamine peptide in hNtaq1. (A) Tripeptides docking study of hNtaq1. Categorized by features of its second residue, the backbone of Ala, Gly, Ile, Leu, Met, Pro, Val tripeptides are colored in yellow, the backbone of Cys, Asn, Gln, Ser, Thr tripetides are colored in green, the His, Lys, Arg tripeptides are colored in blue, the Phe, Trp, Tyr tripeptides are colored in black, and the Asp, Glu tripeptides are colored in orange. (B) The nearest tripeptide in docking study. The substrate-mimicking peptide is shown as green and predicted docking tripeptide Gln-Tyr-Pro is colored in magenta. (C) Binding mode of refined Ser1Gln hNtaq1 mutant on electron density map of hNtaq1. Carbon in substrate-mimicking peptide, catalytic triad, β-strands, loops, and Ser1Gln mutant are colored in green, yellow, cyan, white, and blue, respectively. Oxygen, nitrogen, and sulfur are represented as red, blue, and gold, respectively. Two water molecules are shown as red sphere and electron density map is represented as gray mesh contoured at 2.0 σ.

In order to further elucidate the interaction between hNtaq1 and anticipated substrate peptides with N-terminal glutamine, we conducted docking experiments with all the possible 400 tripeptides with N-terminal glutamine (Gln-X-X), using AutoDock Vina program [23]. Around the binding cleft, there exist three regions that are predominantly negatively-charged, positively-charged, and nonpolar. The docking study was performed to see how the three regions contributes to the recognition of its anticipated substrate. Control docking experiments performed with the three N-terminal residues, Ser-Glu-Gly, showed similar binding poses to that from the crystal structure of hNtaq1 bound with the substrate-mimicking peptide. In the control docking study, stabilization energy of the best predicted binding mode was −4.8 kcal/mol. In the experimental docking study, the average stabilization energy of 400 docking results was 5.0 kcal/mol similar to that of the control docking. From the docking results, most stable tripeptides were QWF, QWW, QWQ, QHW, QWV, QQW, QHF, QGW, QEW, and QWS in the order of stabilization energy ranging from −5.8 to −5.3 kcal/mol. Backbones of all the 400 tripeptides seemed to be recognized by the predominantly negatively-charged patch around the substrate binding cleft, which confers nonspecific interaction with substrates regardless of side chains of second and thereafter residues or with very minor influences (Figure 3A). In the case of the closest tripeptide from the catalytic triad, Gln-Tyr-Pro, C_δ_ of glutamine residue is located 3.15 Å away from sulfhydryl group of Cys28 in the catalytic triad and stabilization energy was −4.6 kcal/mol (Figure 3B).

Interestingly, when we refined our structure with N-terminal Ser1Gln mutation to get a clue of reaction mechanism, modified first serine residue and water2 exactly overlapped with mutated glutamine in the refined structure, the actual substrate of hNtaq1, thus they are reminiscent of an actual substrate (Figure 3C). Sulfur of catalytic Cys28 is 2.8 Å away from the C_δ_ of Gln1s and amide nitrogen of Gln1s is 3.0 Å away from the spot on His81, optimal distance for protonation of a leaving amine group. Carbonyl oxygen of Gln1s seems to lock the N-terminal glutamine via a hydrogen bond to peptide carbonyl of Tyr80 (2.6 Å). We suggest that coordinates of Ser1 and water2 in our crystal structure mimic the pose of an N-terminal glutamine and this binding mode would represent the substrate binding step in hNtaq1 mechanism.

### Proposed mechanism of hNtaq1

**Figure 4:**
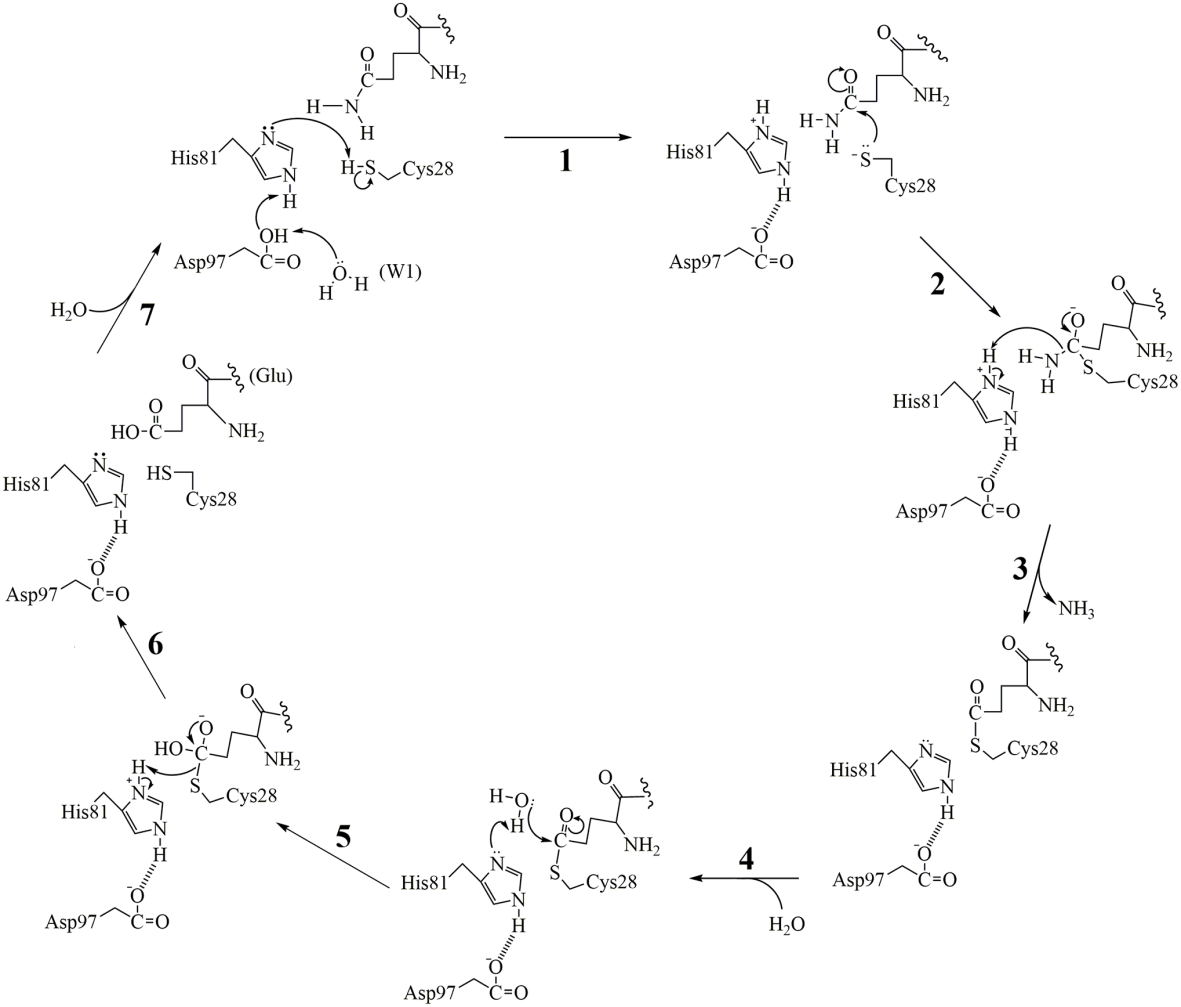
Proposed catalytic mechanism of hNtaq1.

Based on our crystal structure of hNtaq1 and docking study with all the possible anticipated substrate tripeptides, we suggest a catalytic mechanism of hNtaq1 as shown in Figure 4. In the first step, nucleophilic sulfhydryl group of Cys28 approaches C_δ_ of the amide group of the N-terminal glutamine and becomes deprotonated by His81 as shown in Figure 4 step 1. The sulfhydryl group of Cys28 plays a crucial role in the nucleophilic attack on acyl group in the N-terminal glutamine side chain of substrates, which results in formation of a tetrahedral intermediate (Figure 4 step 2). Asp97 facilitates the process by forming a hydrogen bond and electrostatic interactions with His81. The ammonia is released upon productive collapse of the tetrahedral intermediate and a water molecule enters the active site cavity and attacks S-acyl intermediate to convert glutamine to a glutamate (Figure 4 step 3 and 4). As the final step, the glutamate side chain is cleaved from S-acyl of Cys28 (Figure 4 step 5 and 6). In these steps, His81 first acts as a general base activation water for a nucleophilic attack on the S-acyl intermediate, and then upon collapse of the tetrahedral intermediate acts a general acid to protonate the leaving group, i.e. the thiolate of Cys28. The substrate peptide with newly formed N-terminal glutamate is released from the binding cleft at this stage and the enzyme is ready for another round of catalysis (Figure 4 step 7). The proposed reaction mechanism of hNtaq1 and structural information from our study will provide valuable information for understanding the N-end rule pathway and the interaction between hNtaq1 and its protein substrate.
